# Utilizing technology: A cross-sectional study on ICT in healthcare in Kericho County, Kenya

**DOI:** 10.30699/fhi.v12i0.479

**Published:** 2023-09-09

**Authors:** Cyrus T. Tareh, Rose J. Kosgei, Elisha O. Opiyo

**Affiliations:** 1 Department of Biological Sciences, University of Kabianga, Kericho, Kenya; 2 Department of Pediatrics and Child Health, Faculty of Health Sciences, University of Nairobi, Nairobi, Kenya; 3 Department of Computing and Informatics, University of Nairobi, Nairobi, Kenya

**Keywords:** Uptake, Information and Communication, Technology, Healthcare, Health Information, Health Management

## Abstract

**Introduction:**

The potential for Information and Communication Technology (ICT) in healthcare is immense, revolutionizing the delivery of medical services and improving patient outcomes. ICT efficiently manages health information, facilitating electronic health records (EHRs) and streamlined communication among healthcare professionals, leading to significant changes, especially in underserved areas.

**Material and Methods:**

This cross-sectional study took place between March and April 2023 among healthcare professionals in Kericho County, Kenya. Participants were selected using simple random sampling and completed a self-administered questionnaire. Data on the ICT status of health facilities were collected using a checklist. The qualitative component involved key informant interviews with a health record and information department officer. Collected data were entered into Excel and analysed using R software for quantitative data and thematic analysis for qualitative data.

**Results:**

The study engaged 201 participants. Findings showed a 67.66% [95% CI=0.607, 0.741]; p-value<0.0001, uptake of ICT among healthcare workers. Those with computer training were approximately 10 times [OR = 10.867, 95% CI=3.121, 40.99] more likely to utilize ICT in service delivery than those without IT training. Operating at least one healthcare database was associated with over 2 times [OR=2.552, 95% CI=0.7475, 8.7195] higher likelihood of ICT uptake compared to those without this skill. Health facilities with eHealth platforms showed, on average, 38% higher [OR=1.386, 95% CI=0.7661, 2.223] utilization of ICT than those without.

**Conclusion:**

IT training for personnel is crucial, ensuring they can operate preferred health management and information systems (HMIS) within the sector. The presence of an IT department and the use of ICT for administrative purposes significantly affected the general uptake of ICT in health facilities. Additionally, infrastructure such as roads, power, and security had a significant association with ICT compliance. Improving these supportive elements will considerably enhance ICT uptake in healthcare.

## INTRODUCTION

The growth of information and communication technology (ICT) has gone up in recent years due its utilization in life changing transactions [1, 2]. Despite such importance, the ICT uptake and utilization that has been of concern has experienced challenges [3]. Of these challenges, unprecedented growth in population has put a lot of strain on healthcare as observed in unsettling geographical differences cast along various demographic and epidemiological shifts that require persistent spatio-temporal management of healthcare resources [4]. However, ICT can fill this gap by leveraging low cost innovations and low-priced technologies that can provide efficient solutions to some problems in the healthcare sector, thus promoting access to healthcare and improving service delivery.

Luckily, low-cost and sustainable ICT solutions for health – that can augment capacity and improve the overall efficiency – are available [5, 6]. However, the utilization of such ICT tools in healthcare in developing countries still remains low [7]. According to World Bank Group [2], internet access in developing countries remains prohibitively expensive due to a lack of ICT infrastructure. Further, it contends that in the last two decades, the general uptake of ICT has significantly gone up, but the African continent lags well behind other developing regions in internet access and usage. In Kenya, ICT plays the key midwife role for the country’s Vision 2030; the government launched Digital Literacy Program (DLP) to transition young learners to today’s digital world [8]. Consequently, health IT also gained, and continues to gather momentum with the rolled-out eHealth strategy. Despite such success, the uptake and utilization of ICT has been, in part, trying because of the separate sourcing of ICT needs within the same department. This has led to systems that often confuse in integrated decision-making [9], because of a lack of standardization that usually pave way for data disintegration. These challenges can be addressed by advocating for consistency in community-based care systems which open doors for uptake of low-cost technologies that improve quality and accessibility of healthcare services [10].

Presently, the health care sector in Kenya is still largely utilizing tiresome and counterproductive means to handle health related data. Electronic alternatives remain underutilized despite its projected revolutionizing capabilities in data management [7]. Though Kenya enjoys benefits of a firm IT base as well as an elaborate network of satellite communication technology, the application of ICT [11], especially in healthcare, is still at a nascent stage. ICT bolsters the benefits of eHealth in the various areas of medicine since it has the potential of improving service delivery and efficiency in healthcare. While there have been challenges in its acceptance [12], the progression of its uptake is not fully determined. Undoubtedly, the attainment of the goals of universal health coverage is dependent on state of ICT. Therefore, in order to establish a strong and effective primary healthcare system, ICT must play a central role: to support, improve, and speed up the accessibility of healthcare services during service delivery.

## MATERIAL AND METHODS

### Study design, setting, and period

This research utilized a cross-sectional study design conducted during the period of March through April 2023. The study was carried out among healthcare professionals in Kericho County, Kenya. Kericho County is one of the 47 counties established in Kenya following the enactment of the country's Constitution in 2010. It is situated in the southern part of the Rift Valley region in the western part of Kenya, and it is approximately 264 km from Nairobi, the capital city of Kenya. In addition, according to 2019 Census, the county is home to about 901,777 people [13].

Health is a devolved function under the Constitution of Kenya 2010 [14]. Therefore, development in health sector is at different levels in the 47 counties. In order to improve service delivery, public hospitals in Kenya are increasingly purchasing systems to support administrative functions [15].

### Study population

The study population in this study constituted about 2,084 health care workers [16] in all the 366 health facilities in the county who were available, and willing, to participate during data collection period.

### Sample size determination and sampling procedure

The following formula was used:

$$n=\frac{Z^{2}*\;p(1-\;p)}{d^{2}}$$

Where: n=the desired minimum sample size; Z=standard normal deviation (1.96) at 95% confidence level; p=Estimate for p that was based on the Dalberg Report [17], where digital infrastructure was reported to be at 84% in 2021; and d=desired degree of accuracy (0.05). Finally, 207 healthcare workers were considered in this study. In addition, 122 health facilities were assessed for ICT compliance in the study area. Later on, and for the purpose of this study, the six sub-counties were considered as strata and, therefore, the first sampling procedure was stratified sampling method. Simple random sampling was then utilized for the selection of required proportion of the HCP and health facilities across the six sub-counties. For qualitative study, key informant from county health records and information office (CHRIO) was interviewed to provide vital information on the general state of ICT in the county.

### Data collection

The structured self-administered questionnaires used in this study were developed based on a thorough review of relevant literature [18, 19]. To ensure the questionnaire's validity and effectiveness, items in the data collection tool were evaluated by a panel of experts for relevance and clarity. For reliability test, Cronbach's alpha coefficient was calculated to quantify the extent to which the items were interrelated. This was achieved through a pretest on approximately 5% of the total population outside the study area. The questionnaire consisted of two main sections: socio-demographic information and basic IT skills and the utilization of health-based information databases. To assess participants' level of ICT knowledge, six questions related to ICT were included. Additionally, participants were asked about their utilization of ICT, and the questions in this section were rated on a scale of poor, adequate, and excellent to determine the level of ICT usage.

### Study variables

The study focused on examining the relationship between the dependent variable, ICT uptake, and several independent variables. The independent variables included sex, age, academic level, previous IT training status, years in service, the status of infrastructure (such as roads and availability of electricity), and IT knowledge of health-based database.

### Statistical analysis

Data entry was conducted using Excel, while data analysis was performed using R version 4.2.2 software. Descriptive statistics were computed to summarize the socio-demographic characteristics of the participants. Bivariate and multivariable logistic regression analyses were employed to identify factors associated with ICT utilization. In the multivariable logistic regression, variables from the bivariate analysis with a p-value less than 0.05 were included to control for potential confounding effects. The results were interpreted using odds ratios (OR) with a 95% confidence interval (CI) to assess the strength and direction of associations.

## RESULTS

### Reliability and Validity of the study

The results for the consistency and stability of the measurement instrument indicated a high level of reliability, with a Cronbach's alpha coefficient of 0.87, indicating strong internal consistency among the items. Content validity assessment was also conducted with a panel of subject matter experts, consisting of County Health Records and Information Officer (CHRIO), nursing officer, clinical officer, administrative personnel, and a laboratory technologist, evaluated the items for relevance and clarity. The results of the content validity analysis demonstrated a high level of agreement among the experts. Out the items assessed, 85% were rated as 'highly relevant' and 'clear' by the experts. Additionally, valuable feedback from the experts led to minor refinements in certain items.

### Socio-demographic characteristics of study population and ICT competence

The study consisted of a predominantly female participant group, with 105 individuals (52.24%) identifying as female, while 96 individuals (47.76%) identified as male. The majority of participants, accounting for over half of the sample (50.75%), fell within the age range of 26 to 35 years. Those below the age of 25 years constituted the second-largest age group, comprising 23.38% of the participants. In contrast, respondents aged over 46 years were the least represented, making up only 8.46% of the sample. On academics, more than three-quarters (76.62%) of the study participants were holders of diplomas. Certificate (13.43%) and degree (9.95%) holders were second and third, respectively. Also, data was collected across a number of specialized groups in the healthcare. The majority of the professionals who participated in this study were nurses (30.35%), and were followed by those from the health records and information department at 18.41%. Clinicians and public health officers came in third and fourth, respectively. Also, in this study, findings show that over 94% of the healthcare professionals (HCP) are computer-literate. Further, about 9 out 10 (86.07%) HCP had some form of formal computer training. In relevance of work place ICT skills, about two-thirds, 67.66% [95% CI = 0.607, 0.741], p-value = <0.0001, of the participants mentioned that they had adequate knowledge to enable them deliver their services efficiently. This is further supported by another finding in the study whereby the majority, 70.65%, [CI = 0.6383, 0.7684], of the HCP could operate HMIS. Of the named HMIS, Dormax (about 27%) was the most popular platform among the HCP. As revealed in this study, Pepfar and Comprehensive Care Centre (CCC) (each 2.00%) were not as widely used as other systems. Further, 121 facilities were assessed for ICT compliance against a projected figure of 122 that had been proposed. This represented a turnover rate of 99.18%. For qualitative data collection, a CHRIO was interviewed based on the items in the key informant guide and analysis of the transcript done thematically. The demographic findings of the study have been summarized in Table 1.

### Factors associated with ICT uptake

When assessing personnel knowledge and skills of ICT in the multivariable logistic regression model, IT training was the only statistically significant variable. The odds of ICT uptake by a diploma holder were over two times [OR=2.113, 95% CI=0.2335, 23.14] higher than that of a certificate holder. However, this finding was not statistically significant. But, personnel who had some computer training were 10 times [OR=10.87, 95% CI=3.121, 40.99] more likely to utilize ICT in service delivery as opposed to those who had no form of training of IT skills. Moreover, being able to operate at least one of any form of healthcare database was associated with over 2 times [OR=2.552, 95% CI=0.7475, 8.720] more likelihood for ICT uptake unlike those who had no skill on any health database. Further, other factors held constant, literacy levels went up about 4 times [4.726, 95% CI=0.8301, 30.48, p-value=0.079] with more years a health professional served. On assessing secondary factors, such as condition of the road, availability of electricity, and security status, affecting utilization of ICT by the health facilities, security was the only significant variable. Lapse in security was associated with over 66% [0.3363, 95% CI=0.177, 0.517, p-value<0.0001] of underutilization of ICT in healthcare.

On compliance of health facilities for ICT uptake, availability of IT department, eHealth facilities, and any form of health management information system (HMIS), and use of ICT for administrative reasons were statistically significant. A health facility was approximately 61% [OR=0.3911, 95% CI=0.2308, 0.5721] less likely to be considered ICT compliant if only its administrative wing had IT facilities. Another observation was that facilities with IT departments had roughly 1.3 times [OR=1.369, 95% CI=1.056, 1.730] higher chances of utilizing ICT unlike those without. In addition, facilities with eHealth platforms were, on average, utilizing ICT 38% more [OR=1.386, 95% CI=0.7661, 2.223] than those without it. The findings on utilization of ICT in health facilities is summarized in Table 2.

## DISCUSSION

The concept underlying this study was conceived at the backdrop of challenges that were experienced during the Covid-19 pandemic; many patients who had not contracted the flu could not access in-person medical services for fear of getting it at the health facilities. Given the status of eHealth integration is unclear, this study scrutinized the state of uptake and utilization of ICT in healthcare sector. The study found that the ICT uptake among healthcare workers was 67.66% [95% CI=0.607, 0.741]. This finding was higher than that reported by Gagnon and others (54.7%) based on elements that were considered as facilitators for m-health adoption. This gap, therefore, could be because of uptake that has taken place since 2016 when Gagnon reported [20]. Here, we also found that HCP who had training on ICT elements were more likely to utilize the technology during the course of service delivery. This mirrored a number of observations where there had been encouragement by the management [21], for instance, in healthcare sector, to have workforce utilize ICT tools in service delivery. Workshops and trainings have been carried out from time to time and this has increased the number of HCP who are ICT competent and ready to exploit its tools for effective service delivery.

Clearly, there are more diploma holders in healthcare (76.6%). In Kenya, tertiary education is becoming expensive. And while many students perform well in their O-levels, many opt for diploma programs. This is a well calculated compromise because, with diploma, they get to the job market earlier than their degree counterparts. They can then advance their studies once employed and the longer they serve, the more they improve their skills. The study findings revealed that computer literacy levels went up considerably as years in service increased. In experiential learning theory, conclusions lead on those individuals with longer years of service had more exposure and hands-on experience with technology, allowing them to navigate digital environments more efficiently and adapt to new equipment [22].

There is an increase of ICT incorporation in service delivery in many government institutions. In Kenya, the government is actively digitizing its services and over 5000 services are now available on the e-government platform – e-Citizen [23]. This explains why hospitals are also getting ICT facelifts. In the course of this study, two Sub-County – Soin-Sigowet and Roret – hospitals were being fitted with ICT tools. The Kericho County Government is providing ICT infrastructure for its healthcare facilities, utilizing the benefits of fiber optics for prompt IT services. The adoption and use of ICT in health facilities is, in part, credited to optimal user involvement with increased availability of IT infrastructure and systems in health facilities [24]. However, the integration and interoperability of different health management and information systems was not observed in the study [25]. In fact, the CHRIO officer interviewed revealed that the implementation of ICT in healthcare facilities in Kericho County operates as stand-alone nodes but with capabilities of being linked in future. HMIS systems spanning hundreds of health facilities, and a large geographical area, can be expensive to install in a short period of time.

Before all health facilities in the study area get fitted with ICT tools, the local government has been working on supportive infrastructure. Maintenance of feeder roads, and electricity connectivity hitherto to previously remote places has been in progress since the inception of devolution in 2014. This finding was supported by research on contributions of rural transport in achieving sustainable development goals [26] where ICT services and technologies followed the path with better transportation infrastructure. Further, well-kept roads enables the laying down of communication infrastructure such as fibre optic cables [27]. Multivariate analyses of our study revealed positive correlation between uptake of ICT in healthcare and roads, and availability of power; the two most important infrastructure that supports utilization of ICT [28, 29].

### Limitations of the study

The study did not examine the aptitude and attitude of healthcare professionals towards ICT, factors that can significantly influence their computer knowledge and utilization. Furthermore, the information gathered in the study was based on self-perceptions, introducing the possibility of reporting bias. Additionally, the cross-sectional design of the study limits the ability to infer causality between the independent and dependent variables.

## CONCLUSION

There are several variables that affect how well ICT initiatives are implemented and run in healthcare facilities. The accessibility and usability of IT systems have a big impact on how widely adopted and used they are. The efficiency of ICT services is increased through improving IT infrastructure, including hardware, software, and networking capabilities. The expansion of ICT infrastructure and providing dependable connectivity also depend on a well-maintained road system and access to energy. Overall, optimizing the use of ICT infrastructure and systems, improving IT availability and usability, and ensuring robust infrastructure and cybersecurity measures are crucial for successful implementation and operation of ICT initiatives in health facilities. The study also highlighted challenges faced by county health administrators, such as insufficient staff and hindrances to improving skills. Also, mitigating challenges such as cost, and distance – through providing online alternatives – to intentional pursuit of knowledge contribute to improved computer literacy levels.

## Figures and Tables

**Table 1:** Demographic characteristics of the study (N=201)

Variable	Category/Utility	Frequency	%
Sex	Male	96	47.76
	Female	105	52.24
Age	<25 years	47	23.38
	26-35 years	102	50.75
	36-45 years	35	17.41
	>46 years	17	8.46
Academic	Certificate	27	13.43
	Diploma	154	76.62
	Degree	20	9.95
Specialization	Nurse	61	30.35
	Clinician	31	15.42
	Public Health	23	11.44
	Clerk	16	7.96
	HRIO	37	18.41
	Administrator	13	6.47
	Lab technologist	12	5.97
	Pharmacy	8	3.98
Computer literacy	Yes	189	94.04
	No	12	5.96
Computer training	Yes	173	86.07
	No	28	13.93
Work place ICT skills	Poor	3	1.5
	Adequate	135	67.16
	Excellent	63	31.34
Operate any HMIS	Yes	142	70.65
	No	59	29.35
HMIS used	None	70	34.83
	Dormax	54	26.89
	EMR	35	17.41
	DHIS	17	8.46
	CCC	4	2.00
	Pepfar	4	2.00
	NHIF	17	8.46

**Table 2:** ICT Uptake and compliance in healthcare

Variable	Utility	Estimate	OR [95% CI]	p-value
Sex	No	1	[0.388, 4.619]	0.680
	Yes	1.288		
Age	No	1	[0.371, 13.48]	0.361
	Yes	2.324		
Education	No	1	[0.412, 7.678]	0.341
	Yes	2.065		
IT Training	No	1	[3.121, 40.99]	0.0003
	Yes	10.87		
Operate HMIS	No	1	[0.748, 8.72]	0.1311
	Yes	2.552		
Adm	No	1	[0.231, 0.572]	<.0001
	Yes	0.3911		
eHealth	No	1	[0.766, 2.223]	<.0001
	Yes	1.386		
HMIS	No	1	[0.339, 0.700]	<.0001
	Yes	.5041		
IT	No	1	[1.056, 1.730]	<.0001
	Yes	1.369		
Road	Good	1	[−0.130, 0.02]	0.137
	Bad	−0.0582		
Electricity	Present	1	[−0.439, 1.50]	0.657
	Absent	0.1831		
Security	Good	1	[0.177, 0.517]	<0.0001
	Bad	0.3363		
